# PPARG Could Work as a Valid Therapeutic Strategy for the Treatment of Lung Squamous Cell Carcinoma

**DOI:** 10.1155/2020/2510951

**Published:** 2020-06-01

**Authors:** Shunbin Shi, Guiping Yu, Bin Huang, Yedong Mi, Yan Kang, Julia Pia Simon

**Affiliations:** ^1^Department of Thoracic Surgery, Suzhou Ninth People's Hospital, Suzhou, Jiangsu Province 215200, China; ^2^Department of Cardiothoracic Surgery, Jiangyin Hospital of Southeast University Medical College, Jiangyin, Jiangsu Province 214400, China; ^3^Department of General Surgery, Children's National Medical Center, Washington, DC 20010, USA; ^4^Neuroimaging and Informatics Institute, University of Southern California, California, Los Angeles 90007, USA

## Abstract

Previous studies showed that PPAR-gamma (PPARG) ligands might serve as potential therapeutic agents for nonsmall cell lung cancer (NSCLC). However, a few studies reported the specific relationship between PPARG and lung squamous cell carcinoma (LSCC). Here, we made an effort to explore the relationship between PPARG and LSCC. First, we used mega-analysis and partial mega-analysis to analyze the effects of PPARG on LSCC by using 12 independent LSCC expression datasets (285 healthy controls and 375 LSCC cases). Then, literature-based molecular pathways between PPARG and LSCC were established. After that, a gene set enrichment analysis (GSEA) was conducted to study the functionalities of PPARG and PPARG-driven triggers within the molecular pathways. Finally, another mega-analysis was constructed to test the expression changes of PPARG and its driven targets. The partial mega-analysis showed a significant downregulated expression of PPARG in LSCC (LFC = −1.08, *p* value = 0.00073). Twelve diagnostic markers and four prognostic markers were identified within multiple PPARG-LSCC regulatory pathways. Our results suggested that the activation of PPARG expression may inhibit the development and progression of LSCC through the regulation of LSCC upstream regulators and downstream marker genes, which were involved in tumor cell proliferation and protein polyubiquitination/ubiquitination.

## 1. Introduction

PPARG is a ligand-activated transcription factor belonging to the family of peroxisome proliferator-activated receptors (PPARs) [1], which is widely expressed in many cells and tissues in the human body [2]. PPARG has recently attracted interest as the potential therapeutic target for a variety of malignancies [3]. A number of animal models [4], cell lines [5, 6], and clinical trials [NCT00923949, NCT01199068, NCT01199055] demonstrated that activation of PPAR-gamma impedes lung tumor progression and suggest that PPARG ligands may serve as potential therapeutic agents for nonsmall cell lung cancer (NSCLC), with the emphasis on lung adenocarcinoma [7, 8]. For instance, Ni et al.'s study showed that the activation of PPARG could inhibit the proliferation of EGFR-TKI-resistant lung adenocarcinoma cells and lead to a better survival rate [8].

So far, only a few studies explored the relationship between PPARG and lung squamous cell carcinomas (LSCC) [9, 10]. Kim et al. pointed out that the truncated splice variant of human PPAR gamma1 (hPPARG1(tr)) was strongly expressed in primary LSCC tumorous tissues, and the overexpression of hPPARG1(tr)) could increase the resistance of transfected cells to chemotherapeutic drug- and chemical-induced cell death [10]. However, to our knowledge, no study has systematically studied the role of PPARG in the pathology of LSCC.

To address this issue, we first conducted a meta-analysis to study the gene expression change of PPARG in the case of LSCC. Then, we integrated literature-based pathways and gene set enrichment analysis (GSEA) to study the potential pathways where PPARG could exert influence on the pathologic development of LSCC. Our results may help to understand the potential roles of PPARG in the case of LSCC.

## 2. Materials and Methods

### 2.1. Selection of LSCC Expression Data in Mega-Analysis

For the initial selection, we searched the LSCC expression datasets on gene expression omnibus (GEO; https://www.ncbi.nlm.nih.gov/geo/) by using the keywords “lung squamous cell carcinoma.” Then, we applied the following criteria to filter further: (1) The entry type used in the study was series; (2) The dataset was array expression data; (3) The studies were designed as a comparison between LSCC and healthy controls: and (4) The organism of the dataset was Homo sapiens. Finally, we considered the datasets for which the original data and the corresponding format files were downloadable. Since we calculated the expression using the original data as extracted above, we used the term “mega-analysis” instead of “meta-analysis.”.

### 2.2. Mega-Analysis and Partial Mega-Analysis Models

In order to identify the relation between PPARG and LSCC, we used a mega-analysis to analyze the expression levels of PPARG in the case of LSCC. In our study, results from using both the random-effects model and the fixed-effects model were compared. To determine the heterogeneity of the datasets, between- and within-study variance was calculated and compared. When the total variance Q was no bigger than the expected value of the between-study variances (df), the model sets the ISq (percentage of the within- over between-study variance) to zero. In this case, the fixed-effects model, instead of the random-effects model, will be selected for the mega-analysis. All analyses were performed using Matlab (version R2017a; https://www.mathworks.com/products/matlab.html).

Moreover, we performed a partial mega-analysis to discover the significance of a gene presented in part of the studies/datasets (e.g., 50% of total studies) but not in all datasets, where 50% top studies/datasets were employed for the mega-analysis of a gene. Here, we define the “top datasets” for a gene as these datasets that demonstrate the bigger absolute value of effect size than the rest of the datasets. It should be mentioned that the top datasets for different genes could be different.

### 2.3. Analysis of Influential Factors

To estimate the possible influence of several factors (e.g., study date, country of origin, and sample size) on the gene expression in the case of MI, we conducted a multiple linear regression (MLR) analysis and reported the *p* values for each of these factors.

### 2.4. Construct PPARG-Drive Network and Gene Set Enrichment Analysis

Based on large scale literature data mining, we constructed a diagnostic and a prognostic functional network connecting PPARG and LSCC. In the diagnostic network, we identified the genes that were contra-directionally regulated by PPARG and LSCC. To achieve this goal, we used Pathway Studio (http://www.pathwaystudio.com/) to identify PPARG➔gene relationships and LSCC➔gene relationships with polarity. Each of these relationships has supported by one or two scientific references. Then we identified the overlapped genes within these relationships to construct the PPARG—LSCC diagnostic network. To increase the reliability of the identified network, we limited the genes to these that also demonstrated consistency in terms of their gene expression alteration in the case of LSCC in the mega-analysis. For the prognostic pathway, we followed the same pressure but to identify genes that were downstream targets of PPARG and upstream regulators of LSCC. The reference information supporting the relations identified in these networks was provided in the Supplementary Materials (available here), including the type of the relationship, supporting references, and related sentences from the references where the relationship has been identified.

For these genes within the diagnostic and prognostic networks built above, a gene set enrichment analysis (GSEA) was conducted using Pathway Studio (version 12.1.0.9; http://www.pathwaystudio.com/) against Gene Ontology (GO; http://geneontology.org) and Pathway Studio pathways. The purpose of GSEA was to test the functional profile of the genes involved in the PPARG-driven networks.

## 3. Results

### 3.1. Mega-Analysis Based on the Selected LSCC Expression Datasets

There were 4,643 results shown in the GEO datasets identified by the keywords “lung squamous cell carcinoma”. Then, further filters were set as our criteria. A total of 12 datasets satisfied the inclusion criteria for the mega-analysis, which are listed in Table 1. The studies were distributed in 8 different countries, and the study dates ranged from 2 to 15 years ago, including 285 healthy controls and 375 LSCC cases.

**Figure 1:**
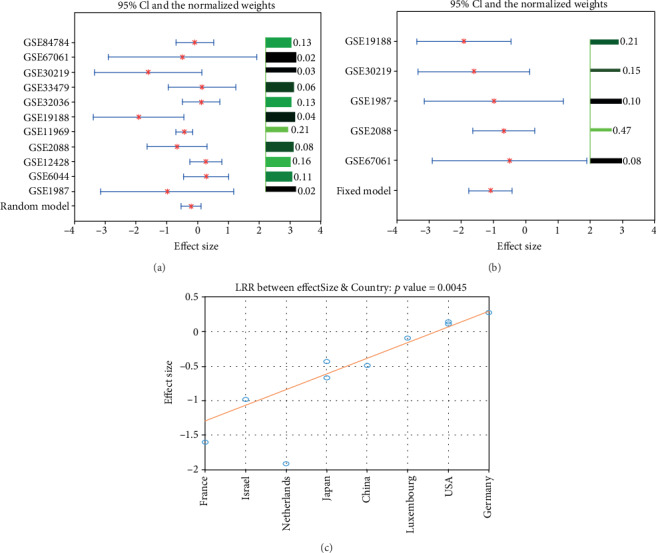
Mega-analysis results of PPARG using 11 LSCC RNA expression datasets. (a) Mega-analysis results from the random-effects model. (b) Partial mega-analysis results from the fixed-effects model. (c) The influence of population region (country) on the PPARG expression levels. The bar plot on the right of each figure represents the normalized weights for each dataset/study, range (0, 1); the brighter (green) the color, the bigger the weight (labeled right next to the bar). The star (in red) and lines (in blue) on the left are the mean of effect size (log fold change) and 95% confidence interval (CI) of each dataset/study, respectively.

The mega-analysis and partial mega-analysis results for gene PPARG are presented in Table 2. As shown in Figure 1(a), the total variance (Q) was larger than the expected between-study variance (*df*), the within-study variance percentage (*ISq*%) was 45.10, the between-study variances were significant, and thus a random-effects model was selected for PPARG in the mega-analysis. However, there were no significant between-study variances (*Isq* = 0, Q test *p* = 0.64), see in Figure 1(b). Thus, the fixed-effects model was selected for PPARG in partial mega-analysis. The LFC of the gene was estimated from about half (top 50%) of the selected datasets. PPARG demonstrated significantly lower expression in the case of LSCC (*LFC* = −1.08, *p* *value* = 0.00073).

MLR analysis showed that sample size and study age were not significant influential factors for the expression levels of PPARG among the 11 LSCC datasets (*p* *value* > 0.30). However, the population region (country) was identified as a significant factor that influences the LFC of PPARG in the case of LSCC (*p* *value* = 0.0045, Figure 1(c)). This may partially explain the differential results between the partial mega-analysis and the mega-analysis.

### 3.2. The LSCC Diagnostic Network Interfered by PPARG

**Figure 2:**
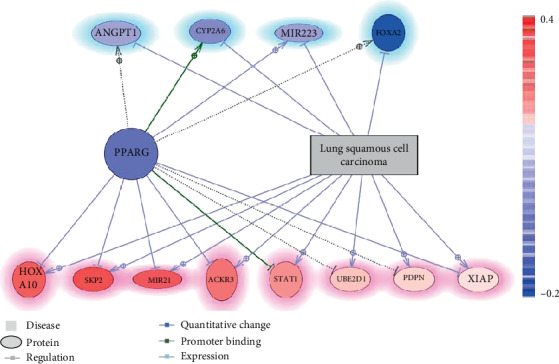
LSCC diagnostic network interfered with PPARG. Genes highlighted in blue (genes at the top of the figure) were literature-implicated with a downregulation in the case of LSCC, and those highlighted in red (genes at the bottom of the figure) were upregulated according to literature reports. Genes in blue represent a decreased expression level from the mega-analysis using 12 LSCC datasets, while those in red represent an increased expression level.

Multiple molecules (12 genes) have been identified through large-scale literature data mining that was contra-directionally influenced by PPARG and LSCC, as shown in Figure 2. According to previous literature reports, a total of 8 molecules (XIAP, UBE2D1, SKP2, ACKR3, MI21, HOXA10, STAT1, and PDPN) were upregulated in LSCC (the genes at the bottom of Figure 2; the arrows with ⊕, Figure 2) but negatively affected by PPARG (the arrows with ┥, Figure 2). These eight genes also presented increased expression levels in the 12 LSCC RNA expression datasets. These results support the literature data mining results and suggest these eight genes as positive markers for LSCC. The mega-analysis results for these genes were provided in the Supplementary Materials→Mega-analysis (available here). The inhibition of these genes by PPARG could exert an anti-LSCC effect during its pathological development (Figure 2).

On the other hand, four genes (MIR223, ANGPT1, CYP2A6, and FOXA2) have been suggested to get suppressed in LSCC (the genes at the top of Figure 2, the arrows with ┥) but were stimulated by PPARG (Figure 2). These four genes also presented decreased expression levels in the mega-analysis, supporting the literature data mining results. Activation of these molecules could be due to other pathways where PPARG inhibits the progress of LA. Detailed information regarding the network presented in Figure 2 can be found in the Supplementary Materials→LSCC (available here) diagnostic network, including the type of the relationships and the supporting references.

### 3.3. GSEA for the Genes Involved in LSCC Diagnostic Network

The GSEA was performed using Pathway Studio with the purpose of investigating the biological functions of the 12 genes within the LSCC diagnostic network. The GSEA was also confirmed by the mega-analysis, including eight upregulated and five suppressed genes. A total of 10 out of these 12 genes were shared among the top 10 most significantly enriched pathways (*p* *values* < 0.012, *q* = 0.05 for FDR), which are presented in Table 3. The full 21 pathways/gene sets enriched with *p* *value* < 0.047 were presented in the Supplementary Materials**→**GSEA1 (available here). Notably, enriched pathways highlighted by the GSEA approach are mainly related to the regulation of protein ubiquitination, the regulation of cell proliferation, and cytokine stimulus.

### 3.4. LSCC Prognostic Network Interfered by PPARG

**Figure 3:**
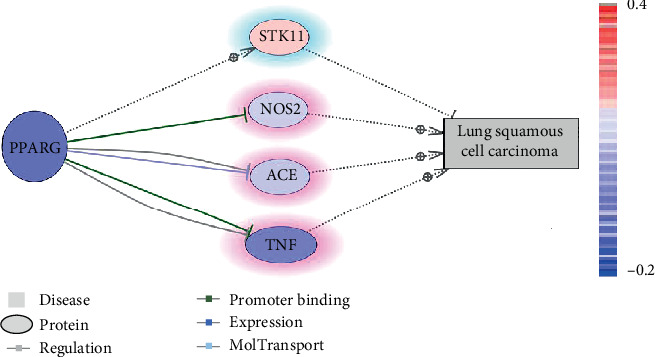
LSCC prognostic network interfered with PPARG. Genes in blue represent a decreased expression level from the mega-analysis using 12 LSCC datasets; entities in red represent an increased expression level. Entities highlighted in blue were literature implicated as the LSCC-inhibitors, while those highlighted in red were LSCC-promoters.

As shown in Figure 3, a regulatory pathway connecting PPARG and LSCC was identified, heavily involved in the pathological development of LSCC. Based on the literature reports, three genes, TNF, NOS2, and ACE, could promote the pathological development of LSCC (highlighted in red, the arrows with ⊕, Figure 3). These three genes were deactivated by PPARG. However, according to the mega-analysis results, these genes were downregulated together with PPARG in the case of LSCC. Therefore, PPARG may not necessarily be needed for the deactivation of these genes to inhibit the progress of LSCC. The supporting references for each relation presented in Figure 3 were provided in the Supplementary Materials→LSCC_ (available here)prognostic network, which also include the type of the relationships.

In addition, an LSCC-inhibitor, STK11, has been shown to be activated by PPARG (highlighted in blue, the arrows with ┥, see Figure 3(a)). Mega-analysis showed that this gene showed slightly increased expression in the case of LSCC. Therefore, activation of PPARG may further promote the activation of STK11, which could be a blocker for the pathologic development of LSCC. The mega-analysis results of these four genes in Figure 3 were provided in the Supplementary Materials**→**Mega-analysis (available here).

### 3.5. GSEA for the Genes Involved in LSCC Prognostic Network

GSEA results showed that the five genes (PPARG, STK11, NOS2, and TNF) were significantly enriched within 59 pathways/gene sets (*p* *value* < 0.044; *q* = 0.05 for FDR; see Supplementary Materials**→**GSEA2 (available here)). We presented the top 10 pathways (4 genes were enriched; *p* < 0.0077) in Table 4. The pathways were mainly related to cell metabolism and hormone level regulation, which was largely different from that of the LSCC diagnostic network. Also, notably, these five genes enrich more pathways than that of the 12 genes within the LSCC diagnostic network, indicating that these five genes were more functionally linked to each other.

## 4. Discussion

Previous studies suggested that the activation of PPARG might be associated with the inhibition of NSCLC [4–6]. However, most studies were focused on the cases of lung adenocarcinoma [4–6]. In this study, we aim to explore the possible linkage between PPARG and LSCC. First, we utilized a mega-analysis and a partial mega-analysis to analyze the potential relationship between PPARG and LSCC. Subsequently, we integrated knowledge from large-scale literature data mining and existing LSCC expression data to construct molecular networks connecting PPARG and LSCC, followed by a GAEA analysis to study the functional profile of the molecules involved in the PPARG-drive network. Our results showed that PPARG was significantly downregulated in about half of the LSCC cases, with multiple pathways suggesting an inhibition role of PPARG in the pathologic development and progress of LSCC.

Notably, PPARG did not show a significant decrease in the 11 studies overall (*LFC* = −0.22; *p* *value* = 0.094), while it demonstrated significant decreased expression in 5 out of the 11 studies (*LFC* = −1.08; *p* *value* = 0.00073). These results indicate that there are influential factors that lead to different expression levels of PPARG among different studies. MLR analysis showed that the population region was a significant factor that influenced the PPARG levels (Figure 1(c)). Specifically, PPARG demonstrated low expression of *LFC* = −1.60 in the dataset from France (GSE30219), while relatively high expression in the dataset from Germany (*LFC* = 0.27; GSE6044). Notably, one of the highest (GSE12428; *LFC* = 0.26) and the lowest (GSE19188; *LFC* = −1.91) expression levels were from the Netherlands, indicating that, besides population region, there could be other factors that influence the PPARG expression levels in LSCC patients. Further investigation showed that the GSE12428 dataset was especially studying LSCC patients who smoke, while the dataset of GSE19188 contained all patients in general. It has been shown that smoking is one of the major risk factors for the development of LSCC [11], and smokers tend to have low PPARG expression levels [12]. These studies may explain the different PPARG levels in these two Netherlands datasets. Moreover, MLR results also suggest that sample size and study date were not significant factors for the PPARG levels. Due to lack of data, we only studied the influence of three factors on the expression levels of PPARG. Further study is needed to test the expression level of PPARG in LSCC patients and the possible influential factors such as age, gender, and complication.

Functional network analysis showed that PPARG could play roles both in the development and progression of LSCC. Specifically, PPARG counter-regulated 12 molecules that were upregulated or downregulated by LSCC (Figure 2). The expression levels of eight LSCC markers (XIAP, UBE2D1, SKP2, ACKR3, MI21, HOXA10, STAT1, and PDPN) were significantly upregulated in LSCC patients [13–20] and were downregulated by PPARG [21–28]. On the other hand, PPARG could activate multiple genes [29–33] inhibited by LSCC [34–38], including MIR 223, PTEN, ANGPT1, CYP2A6, and FOXA2. It should also be noted that the relations between LSCC and the molecules in the network are supported by both the literature data mining and mega-analysis using 12 LSCC expression datasets, which strengthens the reliability of the PPARG-driven network.

GSEA analysis suggested that PPARG may influence the development of LSCC through multiple pathways (Table 3 and Table 4). Besides the regulation of cell proliferation associated with the procession of LSCC, PPARG could also regulate protein polyubiquitination and ubiquitination, which has become increasingly recognized as a controller to regulate the function and signaling of a profusion of proteins. Ubiquitination affects proteins in various cellular processes, including signal transduction, DNA repair, chromosome maintenance, transcriptional activation, cell cycle progression, cell survival, and certain immune cell functions [39]. Thus, it is not surprising that ubiquitin metabolism enzymes prominently feature either oncogenes or tumor suppressors in a variety of cancers and many pathways relevant to cancer. A previous study suggested that targeting those physiological processes may effectively abate the proliferation and facilitate the treatment of lung cancer cells [40]. In the LSCC diagnostic network (Figure 2), E2 ubiquitin-conjugating enzymes (UBE2D1), E3 ubiquitin ligases (XIAP), and SKP2 were involved in the regulation of protein ubiquitination. The expression of XIAP, UBE2D1, and SKP2 downregulated by PPARG at the transcriptional level [21, 22, 41] were overexpressed in LSCC tissues with robust proliferation ability [13–15].

PPARG may also play a role in the progression of LSCC by interfering upstream regulators of LSCC, as shown in Figure 3. For instance, the knock-down of PPARG has been shown to deactivate STK11 [42], while the loss of STK11 could lead to the formation of LSCC [43]. Furthermore, the activation of PPARG could inhibit three promoters of LSCC, including NOS2 [44], ACE [45], and TNF [46]. Thus, increased expression of PPARG may inhibit the formation of LSCC.

The most significant contribution of this study was the identification of the two PPARG-driven networks (Figures 2 and 3) that partially explain the mechanism of the roles of PPARG in the etiology and development of LSCC. However, the integration of literature data-mine and mega-analysis may exclude potential genes/molecules connection PPARG and LSCC. Further study is needed to validate and consummate the networks identified here.

## 5. Conclusion

Results from this study indicated that the expression of PPARG might be suppressed in LSCC patients. Activation of PPARG expression may inhibit the development and progress of LSCC through the regulation of LSCC upstream regulators and downstream marker genes. Our results indicate the need for further study of the relationship between PPARG and LSCC.

## Figures and Tables

**Table 1:** The 12 qualified LSCC expression datasets for mega-analysis.

GEO ID	Control (*n*)	Case (*n*)	Country	Study age	Sample organism
GSE84784	9	9	Luxembourg	2	Homo sapiens
GSE67061	8	69	China	3	Homo sapiens
GSE30219	14	61	France	5	Homo sapiens
GSE33479	27	14	USA	5	Homo sapiens
GSE32036	59	12	USA	7	Homo sapiens
GSE19188	65	27	Netherlands	9	Homo sapiens
GSE11969	5	35	Japan	10	Homo sapiens
GSE2088	30	48	Japan	10	Homo sapiens
GSE12428	28	34	Netherlands	11	Homo sapiens
GSE6044	5	14	Germany	13	Homo sapiens
GSE1987	7	17	Israel	15	Homo sapiens
GSE12472	28	35	Netherland	10	Homo sapiens

**Table 2:** Analysis of PPARG expression levels in LSCC datasets.

PPARG	Mega-analysis	Partial mega-analysis
Models	Random effects	Fixed effects
# study	11	5
LFC (effect size)	-0.22	-1.08
*p* value	0.094	0.00073
ISq (%)	45.10	0.00
*p* value Q	0.051	0.64
# sample	0.85	0.10
Country	0.0045	2.79e-6
Study age	0.30	0.00034

**Table 3:** The top 10 genetic pathways enriched by the 12 genes within LSCC_diagnostic network.

Name	GO ID	Overlap	*p* value	Jaccard similarity
GO: positive regulation of protein polyubiquitination	1902916;	3	0.00085	0.14
GO: regulation of smooth muscle cell proliferation	0048660;	5	0.0012	0.022
GO: regulation of protein polyubiquitination	1902914;	3	0.0015	0.097
GO: positive regulation of protein ubiquitination	0031398;	4	0.0036	0.029
GO: positive regulation of protein modification by small protein conjugation or removal	1903322;	4	0.0050	0.025
GO: regulation of response to cytokine stimulus	0060759;	4	0.0052	0.022
GO: regulation of cytokine-mediated signaling pathway	0001959;	4	0.0052	0.023
GO: regulation of endothelial cell proliferation	0001936;	4	0.0080	0.019
GO: regulation of protein ubiquitination	0031396;	4	0.010	0.017
GO: regulation of response to external stimulus	0032101;	6	0.011	0.0060

**Table 4:** The top 10 genetic pathways enriched by the 12 genes within LSCC_diagnostic network.

Name	GO ID	Overlap	*p* value	Jaccard similarity
GO: positive regulation of small molecule metabolic process	0062013	4	0.00054	0.023
GO: regulation of small molecule metabolic process	0062012	4	0.0036	0.0095
GO: fatty acid transport	0015908	3	0.0036	0.037
GO: receptor biosynthetic process	0032800	2	0.0036	0.22
GO: response to oxygen levels	0070482	4	0.0049	0.0073
GO: regulation of inflammatory response	0050727	4	0.0049	0.0079
GO: cellular response to organic cyclic compound	0071407	4	0.0076	0.0063
GO: regulation of hormone levels	0010817	4	0.0077	0.0060
GO: monocarboxylic acid transport	0015718	3	0.0077	0.019
CSF2 -> STAT expression targets	NONE	4	0.0077	0.055

## Data Availability

The data of this study are available from the corresponding author upon reasonable request.

## Supplementary Materials

Supplementary Materials (1) GSEA1 and GAEA2 present GSEA results for the genes within Figure 2 and Figure 3, respectively. (2) Mega-analysis presents the mega-analysis results for PPARG gene and its driven genes within the Figure 2 and Figure 3. (3) Partial_Mega-analysis presents the partial mega-analysis results for PPARG; (4) LSCC_diagnostic network presents the reference information for the network presented in Figure 2. (4) LSCC_prognostic network presents the reference information for the network presented in Figure 3.

## References

[1] Bocher V., Chinetti G., Fruchart J. C., Staels B. (2002). Role of the peroxisome proliferator-activated receptors (PPARS) in the regulation of lipids and inflammation control. *Journal de la Societe de biologie*.

[2] Berger J., Moller D. E. (2002). The mechanisms of action of PPARs. *Annual Review of Medicine*.

[3] Peters J. M., Shah Y. M., Gonzalez F. J. (2012). The role of peroxisome proliferator-activated receptors in carcinogenesis and chemoprevention. *Nature Reviews Cancer*.

[4] Skrypnyk N., Chen X., Hu W. (2014). PPAR*α* activation can help prevent and treat non-small cell lung cancer. *Cancer Research*.

[5] Keshamouni V. G., Reddy R. C., Arenberg D. A. (2004). Peroxisome proliferator-activated receptor- *γ* activation inhibits tumor progression in non-small-cell lung cancer. *Oncogene*.

[6] Kaur S., Nag A., Gangenahalli G., Sharma K. (2019). Peroxisome Proliferator Activated Receptor Gamma Sensitizes Non-small Cell Lung Carcinoma to Gamma Irradiation Induced Apoptosis. *Front Genet*.

[7] Susaki Y., Inoue M., Minami M. (2012). Inhibitory effect of PPAR*γ* on NR0B1 in tumorigenesis of lung adenocarcinoma. *International Journal of Oncology*.

[8] Ni J., Zhou L. L., Ding L. (2017). PPAR*γ* agonist efatutazone and gefitinib synergistically inhibit the proliferation of EGFR-TKI-resistant lung adenocarcinoma cells via the PPAR*γ*/PTEN/Akt pathway. *Experimental Cell Research*.

[9] Li H., Weiser-Evans M. C., Nemenoff R. (2012). Anti- and Protumorigenic Effects of PPAR*γ* in Lung Cancer Progression: A Double-Edged Sword. *PPAR Research*.

[10] Kim H. J., Hwang J. Y., Kim H. J. (2007). Expression of a peroxisome proliferator-activated receptor gamma 1 splice variant that was identified in human lung cancers suppresses cell death induced by cisplatin and oxidative stress. *Clinical Cancer Research*.

[11] Boelens M. C., van den Berg A., Fehrmann R. S. (2009). Current smoking-specific gene expression signature in normal bronchial epithelium is enhanced in squamous cell lung cancer. *The Journal of Pathology*.

[12] Bergman B. C., Perreault L., Hunerdosse D. M., Koehler M. C., Samek A. M., Eckel R. H. (2009). Intramuscular lipid metabolism in the insulin resistance of smoking. *Diabetes*.

[13] Chen Y. B., Shu J., Yang W. T. (2011). XAF1 as a prognostic biomarker and therapeutic target in squamous cell lung cancer. *Chinese Medical Journal*.

[14] Hou L., Li Y., Wang Y. (2018). UBE2D1 RNA Expression Was an Independent Unfavorable Prognostic Indicator in Lung Adenocarcinoma, but Not in Lung Squamous Cell Carcinoma. *Disease Markers*.

[15] Zhong K., Yang F., Han Q., Chen J., Wang J. (2018). Skp2 expression has different clinicopathological and prognostic implications in lung adenocarcinoma and squamous cell carcinoma. *Oncology Letters*.

[16] Behnam Azad B., Lisok A., Chatterjee S. (2016). Targeted Imaging of the Atypical Chemokine Receptor 3 (ACKR3/CXCR7) in Human Cancer Xenografts. *Journal of Nuclear Medicine*.

[17] Song Y., Dou H., Wang P. (2014). A novel small-molecule compound diaporine A inhibits non-small cell lung cancer growth by regulating miR-99a/mTOR signaling. *Cancer Biology Therapy*.

[18] Clemenceau A., Boucherat O., Landry-Truchon K. (2018). Lung cancer susceptibility genetic variants modulate HOXB2 expression in the lung. *The International Journal of Developmental Biology*.

[19] Yang M., Chen H., Zhou L., Chen K., Su F. (2019). Expression profile and prognostic values of STAT family members in non-small cell lung cancer. *American journal of translational research*.

[20] Suzuki H., Onimaru M., Koga T. (2011). High podoplanin expression in cancer cells predicts lower incidence of nodal metastasis in patients with lung squamous cell carcinoma. *Pathology, Research and Practice*.

[21] Bräutigam K., Biernath-Wüpping J., Bauerschlag D. O. (2011). Combined treatment with TRAIL and PPAR*γ* ligands overcomes chemoresistance of ovarian cancer cell lines. *Journal of Cancer Research and Clinical Oncology*.

[22] Almeida P. E., Carneiro A. B., Silva A. R., Bozza P. T. (2012). PPAR*γ* Expression and Function in Mycobacterial Infection: Roles in Lipid Metabolism, Immunity, and Bacterial Killing. *PPAR Research*.

[23] Meng J., Ding Y., Shen A. (2010). Overexpression of PPAR*γ* can down-regulate Skp2 expression in MDA-MB-231 breast tumor cells. *Molecular and Cellular Biochemistry*.

[24] Zhao D., Zhu Z., Li D., Xu R., Wang T., Liu K. (2015). Pioglitazone Suppresses CXCR7 Expression To Inhibit Human Macrophage Chemotaxis through Peroxisome Proliferator-Activated Receptor *γ*. *Biochemistry*.

[25] Zhang Y. F., Xu H. M., Yu F. (2018). Crosstalk between MicroRNAs and Peroxisome Proliferator-Activated Receptors and Their Emerging Regulatory Roles in Cardiovascular Pathophysiology. *PPAR Research*.

[26] Yasmeen R., Meyers J. M., Alvarez C. E. (2013). Aldehyde dehydrogenase-1a1 induces oncogene suppressor genes in B cell populations. *Biochimica et Biophysica Acta (BBA) - Molecular Cell Research*.

[27] Costa V., Gallo M. A., Letizia F., Aprile M., Casamassimi A., Ciccodicola A. (2010). PPARG: Gene Expression Regulation and Next-Generation Sequencing for Unsolved Issues. *PPAR Research*.

[28] Zhu X., Chen Y., Zhu W. (2019). Oroxylin A inhibits Kaposi’s sarcoma-associated herpes virus (KSHV) vIL-6-mediated lymphatic reprogramming of vascular endothelial cells through modulating PPAR*γ*/Prox1 axis,. *Journal of Medical Virology*.

[29] Ying W., Tseng A., Chang R. C. A. (2015). MicroRNA-223 is a crucial mediator of PPAR*γ*-regulated alternative macrophage activation. *Journal of Clinical Investigation*.

[30] Farrow B., Evers B. M. (2003). Activation of PPAR*γ* increases PTEN expression in pancreatic cancer cells. *Biochemical and Biophysical Research Communications*.

[31] Fink T., Abildtrup L., Fogd K. (2004). Induction of adipocyte-like phenotype in human mesenchymal stem cells by hypoxia. *Stem Cells*.

[32] Deng J., Guo L., Wu B. (2018). Circadian Regulation of Hepatic Cytochrome P450 2a5 by Peroxisome Proliferator-Activated Receptor*γ*. *Drug Metabolism and Disposition*.

[33] Kim H. S., Hwang Y. C., Koo S. H. (2013). PPAR-*γ* activation increases insulin secretion through the up-regulation of the free fatty acid receptor GPR40 in pancreatic *β*-cells. *PLoS One*.

[34] Luo P., Wang Q., Ye Y. (2019). MiR-223-3p functions as a tumor suppressor in lung squamous cell carcinoma by miR-223-3p-mutant p53 regulatory feedback loop. *Journal of Experimental & Clinical Cancer Research*.

[35] Jolly R. D., Thompson K. G., Winchester B. G. (1975). Bovine mannosidosis--a model lysosomal storage disease. *Bovine mannosidosis--a model lysosomal storage disease*.

[36] Yao S., Dong S. S., Ding J. M. (2019). Sex-specific SNP-SNP interaction analyses within topologically associated domains reveals ANGPT1 as a novel tumor suppressor gene for lung cancer. *Genes Chromosomes Cancer*.

[37] Hosono H., Kumondai M., Arai T. (2015). CYP2A6 genetic polymorphism is associated with decreased susceptibility to squamous cell lung cancer in Japanese smokers. *Drug Metabolism and Pharmacokinetics*.

[38] Tang L., Liu J., Zhu L. (2018). Curcumin Inhibits Growth of Human NCI-H292 Lung Squamous Cell Carcinoma Cells by Increasing FOXA2 Expression. *Frontiers in Pharmacology*.

[39] McBride W. H., Iwamoto K. S., Syljuasen R., Pervan M., Pajonk F. (2003). The role of the ubiquitin/proteasome system in cellular responses to radiation. *Oncogene*.

[40] Tang Y., Geng Y., Luo J. (2015). Downregulation of ubiquitin inhibits the proliferation and radioresistance of non-small cell lung cancer cells _in vitro_ and _in vivo_. *Scientific Reports*.

[41] Wang S. T., Ho H. J., Lin J. T., Shieh J. J., Wu C. Y. (2017). Simvastatin-induced cell cycle arrest through inhibition of STAT3/SKP2 axis and activation of AMPK to promote p27 and p21 accumulation in hepatocellular carcinoma cells. *Cell Death Disease*.

[42] Ji J., Xue T. F., Guo X. D. (2018). Antagonizing peroxisome proliferator-activated receptor *γ* facilitates M1-to-M2 shift of microglia by enhancing autophagy via the LKB1-AMPK signaling pathway. *Aging Cell*.

[43] Xu C., Fillmore C. M., Koyama S. (2014). Loss of Lkb1 and Pten leads to lung squamous cell carcinoma with elevated PD-L1 expression. *Cancer Cell*.

[44] Cullingford T. E. (2004). The ketogenic diet; fatty acids, fatty acid-activated receptors and neurological disorders. *Prostaglandins, Leukotrienes, and Essential Fatty Acids*.

[45] Vallée A., Lévy B. L., Blacher J. (2018). Interplay between the renin-angiotensin system, the canonical WNT/*β*-catenin pathway and PPAR*γ* in hypertension. *Current Hypertension Reports*.

[46] Hsueh W. A., Law R. (2003). The central role of fat and effect of peroxisome proliferator-activated receptor–*γ* on progression of insulin resistance and cardiovascular disease. *The American Journal of Cardiology*.

